# Editorial: Getting Neuroprosthetics Out of the Lab: Improving the Human-Machine Interactions to Restore Sensory-Motor Functions

**DOI:** 10.3389/frobt.2022.928383

**Published:** 2022-05-25

**Authors:** Aaron M. Dingle, Karen Moxon, Solaiman Shokur, Ivo Strauss

**Affiliations:** ^1^ Division of Plastic Surgery, Department of Surgery, University of Wisconsin- Madison, Madison, WI, United States; ^2^ Department of Biomedical Engineering, University of California at Davis, Davis, CA, United States; ^3^ École Polytechnique Fédérale de Lausanne, Lausanne, Switzerland; ^4^ Department of Excellence in Robotics and A.I, Scuola Superiore Sant’Anna, Pisa, Italy

**Keywords:** neuroprosthetics, human-machine, peripheral nerve interface, brain-machine (computer) interface, closed-loop, neurorehabilitaiton, clinical translation, neuroscience

In the last decade several neuroprostheses have reached technical maturity and are now used in patients’ daily activities in their homes, including, for example, osseointegration with peripheral nerve interfaces (PNI) to control a robotic prosthesis for patients with amputation (Ortiz-Catalan et al., 2020). As neuroprosthetics continues to mature, we see an increasing number of successful technologies making the transition from bench to bedside. This is particularly true in the BCI space in recent years, where we see increased clinical translation of neurorehabilitation and monitoring devices to manage conditions like tetraplegia and stroke or epilepsy respectively (Cook et al., 2013; Irimia et al., 2016; Opie and Oxley, 2019; Simeral et al., 2021). Today, BCI devices have obtained regulatory approval (Cook et al., 2013; Irimia et al., 2016; Opie and Oxley, 2019), but also (and potentially more importantly) patient and clinician acceptance. Since devices are covered by public and private health insurers, inevitably, a stable product market has developed. On the other hand, many neuroprosthetic devices are still in their proof-of-concept stage. The collection of articles on the current Research Topic identifies key aspects of neuroprosthetic research providing examples and guidance toward delivering state-of-the-art neuroprosthetic technology to a broad range of patients.

A key theme presented throughout this Research Topic has been multidisciplinary collaboration, a theme strongly supported by the editors (Shokur et al., 2021; Karczewski et al., 1102). As technologies move from the bench to the bedside, there is an increased need for collaboration between academic researchers and clinicians, as well as industry and government partners and, of course, the patients. With the here presented research topic we want to encourage the collaboration between the before mentioned entities. It is of great importance to have an overview of the steps it takes from fundamental research to the final product.

Patients living with neurological deficiencies represent key stakeholders of neuroprosthetic technologies, and therefore serve as invaluable members of many clinical research teams. Much of what we know about prosthetic usage comes from acute evaluations in rehabilitation clinics or heavily controlled laboratory settings. Given the increasing maturity of these technologies, research is now expanding to include long-term studies in users’ homes to study realistic their daily needs. Towards user-centered prosthetics research beyond the laboratory (Jones et al.) provides a framework for collaboratively establishing needs-based assessments to facilitate relevant in-home research. Through the engagement of multiple stakeholders, the challenges and opportunities of clinical research in private homes were weighed against the potential to address users’ real-world needs. Clinical research in private homes present many challenges in particular from data privacy, and policy perspectives. It, is also, of great importance to clinicians for improving the functionality and, ultimately user satisfaction since the usage of neuroprostheses at home often identifies new challenges compared to a clinical setting.

Neuroprostheteses replacing daily functions need to last decades without creating cognitive or computational burdens for the user. Increasing robustness of brain-computer interfaces through automatic detection and removal of corrupted input signals (Vasko et al.) utilizes Statistical Process Control frameworks to identify corrupt neural recording channels in a neural network decoder. Corrupted channels are adjusted without requiring user input. The ability to compensate for corrupted BCI data has the potential to reduce computational and user burden while also improving the robustness and longevity of the BCI required for chronic uses in everyday living.

Standardization is another fundamental step when considering very long-term protocols. Challenges and opportunities for the future of brain computer interface in neuromodulation (Simon et al.) provides an overview of the present capacity and future potential for clinical application of BCIs to treat a range of neurological deficiencies. Limitations in learning exist for patients and BCI devices. Clarification of neural mechanisms relative to motor neurorehabilitation was identified as a key component for improving clinical applications of BCI, including exploration of the interplay between cognitive and motor domains relative to the quality of life. Continued multidisciplinary collaboration between fundamental and clinical research is required to drive mechanism-based application alongside standardization of guidelines and protocols to facilitate improved clinical outcomes.

Most of neuroprosthetic development is performed in rodents, often serving as of proof of concept (Aman et al., 2020). Neuroprosthetics with the potential for clinical translation is often tested in larger animals to determine chronic safety and efficacy and ultimately enter human clinical trials. Anatomical pre-clinical studies are far less common in the literature, but can provide integral implications for neuroprosthetic design and application, particularly for more invasive procedures (Karczewski et al.). Clinical basis for creating an osseointegrated neural interface utilizes anatomical dissection and virtual implantation to demonstrate the clinically relevant framework to create an osseointegrated neural interface (ONI) for prosthetic control. The ONI represents a modular surgical strategy for neural interfacing, combining clinical treatment for post-amputation pain with osseointegration, and capable of utilizing a range of neural interfaces based on the site of amputation and patients’ needs. Implementation of novel osseointegrated implants was used to demonstrate the need to remove less bone and increase intramedullary free space for the housing of the neural interface components. While this technique and technology remain experimental, the use of clinically relevant techniques applied to human anatomy address key technical aspects required for clinical and regulatory acceptance towards clinical trials and translation.

Finally, Turning neural prosthetics into viable products (Loeb and Richmond) provides a comprehensive account of the paths and processes required to take technologically complex and invasive BCIs to the market. This includes regulatory requirements of the North American Food and Drug Administration (FDA) and European health, safety and environmental protection standards (CE). Furthermore, considerations for intellectual property, risk management and potential for medical reimbursement must be made. The presented roadmap highlights the importance of collaborations between academia, industry and the government in delivering improved quality of life to patients living with neurological deficiencies.
